# Measurement of Indoor Thoron Gas Concentrations Using a Radon-Thoron Discriminative Passive Type Monitor: Nationwide Survey in Japan

**DOI:** 10.3390/ijerph18031299

**Published:** 2021-02-01

**Authors:** Tetsuya Sanada

**Affiliations:** Department of Radiological Technology, Faculty of Health Sciences, Hokkaido University of Science, Sapporo, Hokkaido 006-8585, Japan; sanada-t@hus.ac.jp

**Keywords:** thoron, radon, indoor, radioactivity, environment, nationwide survey, SSNTD

## Abstract

As part of a nationwide survey of thoron (^220^Rn) in Japan, the indoor ^220^Rn gas concentrations in 940 dwellings were measured throughout one year, from 1993 to 1996, using a passive type ^222^Rn-^220^Rn discriminative monitor. The monitor was placed in a bedroom or a living room in each house for four successive three-month periods. The mean annual indoor ^220^Rn concentration was estimated from the four measurements in each house. The arithmetic mean, the median and the geometric mean for indoor ^220^Rn concentrations in 899 dwellings were 20.1, 9.6 and 10.0 Bq m^−3^, respectively. The ^220^Rn concentrations exhibited a log-normal distribution. It was found that the ^220^Rn concentrations were dependent on the nature of the materials used for wall construction and also on the distance of measurement from the wall. Significant seasonal variations in the ^220^Rn concentration were not observed. It would seem that the nature of the wall material contributed to the increased indoor ^220^Rn concentrations.

## 1. Introduction

Radon (^222^Rn), thoron (^220^Rn) and their progeny species are large contributors to the annual exposure of an effective dose to the general population. ^222^Rn and its progeny species contribute about half of the annual effective dose due to natural radiation based on the world mean dose. According to the United Nations Scientific Committee on the Effects of Atomic Radiation [1], the annual effective dose from natural radiation sources has been calculated to be 2.4 mSv as the worldwide average, whereas ^222^Rn and ^220^Rn contribute 1.2 and 0.1 mSv, respectively. ^222^Rn and ^220^Rn are products of the decay chains of natural radionuclides, such as the ^238^U and ^232^Th series, and have half-lives of 3.825 days and 55 s, respectively. The ^220^Rn half-life is very short compared with ^222^Rn. Thus, only a very small amount of ^220^Rn can enter a room from the outside. It is considered that a ^220^Rn concentration gradient exists near the mud-based walls and floors in low ventilated houses [2]. Therefore, if a mud mortar wall is present in housing materials which have high concentrations of thorium, ^220^Rn and its decay products may enter houses and cause potential health problems. In particular, traditional wooden houses with mud mortar walls are a common house type in Japan.

The International Commission on Radiological Protection (ICRP) [3] have issued new dose conversion factors for ^222^Rn and ^220^Rn progeny species based on a dosimetric approach in Publication 137. The values specified are 16.8 and 107 nSv (Bq m^−3^ h)^−1^, respectively. This means that even small amounts of ^220^Rn progeny species will cause higher radiation exposure compared to ^222^Rn [4]. Therefore, interest in ^220^Rn exposure is growing among the health sciences communities. Recently, a number of ^220^Rn surveys have been carried out in local regions and nationwide, and the results have been published enabling an evaluation of exposures from ^220^Rn [5,6,7,8,9,10,11,12,13,14,15,16,17,18,19,20,21]. Also, the need to adopt reliable ^220^Rn measurement techniques has been argued in several papers [22].

An indoor ^222^Rn survey was conducted on 940 houses nationwide in Japan from 1993 to 1996 using ^222^Rn–^220^Rn discriminative passive type monitors [23]. The passive monitor, developed by Doi and Kobayashi [2], was placed in either a bedroom or a living room where residents spent most of their time. Indoor ^222^Rn concentrations were determined at 20 dwellings in each prefecture for four successive three-month periods to cover an entire year. In the survey, to eliminate the influence of ^220^Rn on ^222^Rn measurement, the ^220^Rn concentration was performed at the same time for referencing purposes. The ^222^Rn and ^220^Rn calibration experiments were performed in a standard radon chamber at the National Radiological Protection Board (Didcot, UK) and using the ^222^Rn–^220^Rn mixed chamber of Waseda University (Tokyo, Japan), respectively. This study is concerned with the results for the indoor ^220^Rn concentrations using the reference data from the nationwide survey which was conducted to determine the ^222^Rn concentrations in Japan [23]. Furthermore, the seasonal and regional variations were investigated, and the influence of the type of house structure was examined as mentioned previously. However, this study does not include a dose assessment of ^220^Rn because the ^220^Rn concentration varies widely in rooms and it is not easy to measure the activity concentration given the short half-life of the radioisotope [22].

## 2. Materials and Methods

### 2.1. ^220^Rn Monitor and Measurement Periods

The solid-state nuclear track detector (SSNTD) was developed at the National Institute of Radiological Sciences (Chiba, Japan) as a ^222^Rn and ^220^Rn discriminative monitor [2]. The monitor consists of two electroconductive hemispheres and there are two polycarbonate films installed in the center of the two hemispheres. To isolate and separate the progeny species of ^222^Rn and ^220^Rn, a glass fiber filter is located in the first hemisphere. Therefore, only gaseous ^222^Rn and ^220^Rn can pass through the filter and enter the first hemisphere. This monitor has two different diffusion chambers which have relatively large and small ventilation rates. This system has been developed based on the large difference in half-lives of ^222^Rn and ^220^Rn. After being exposed, the film was first subjected to chemical etching with a mixed solution of 8 mol L^−1^ KOH and 20% C_2_H_5_OH at 30 °C for 30 min [23]. Then the films were electrochemically etched at 800 V and 2000 Hz for 2 h. A control film, which was exposed to particles from an ^241^Am source and which had been etched simultaneously with the sample films, was also prepared to assure the stability of the etching condition. The track density was converted to the average ^220^Rn concentration by the calibration factor after subtraction of the background track density, i.e., 3.5 ± 1.8 tracks cm^−2^. In the case of the three month long exposure period, the detection limit (DL) for the concentration of ^220^Rn was estimated to be 7.4 Bq m^−3^ (k = 1.65), the definition of DL being based on the definition of Currie [24]. Four monitors were used in the survey to determine the mean annual ^220^Rn concentration. Consequently, the DL for the mean annual ^220^Rn concentration was estimated to be about 1/2 of DL value specified above. The measurements were carried out for four successive three-month periods to cover a whole year (i.e., January–March, April–June, July–September and October–December) for estimation of the mean annual indoor ^220^Rn concentration. The survey was carried out for four years (January 1993–June 1996) and conducted in the same manner as reported previously [23].

### 2.2. ^220^Rn Calibration Experiments

The ^222^Rn and ^220^Rn calibration experiments were performed in a standard radon chamber at the National Radiological Protection Board in the UK and at the ^222^Rn–^220^Rn mixed chamber of Waseda University, Tokyo, respectively [25]. ^220^Rn conversion factor was evaluated to be 0.0098 ± 0.0016 (tracks cm^−2^ per Bq m^−3^ d).

## 3. Results and Discussion

### 3.1. Distribution of ^220^Rn Concentration

**Figure 1:**
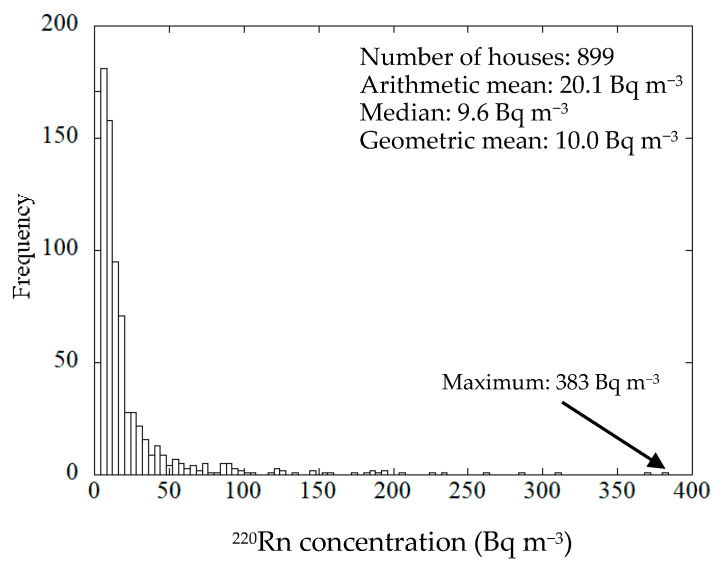
Histogram for indoor ^220^Rn concentrations.

**Figure 2:**
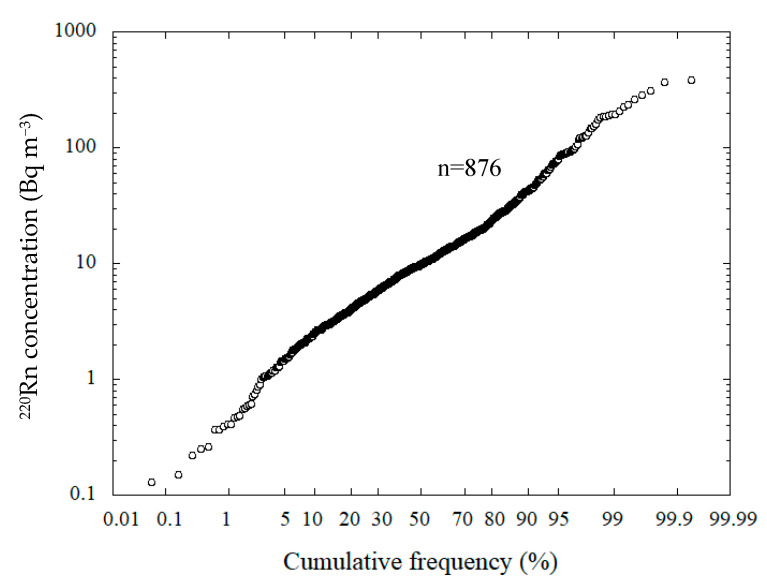
Cumulative frequency distribution for indoor ^220^Rn concentrations. This figure has been prepared using the mean annual ^220^Rn concentrations in excess of zero Bq m^−3^.

The mean annual ^220^Rn concentrations were obtained for 899 houses, the number of houses monitored being reduced from the original 940 houses as was the case for ^222^Rn [23]. The annual arithmetic mean, and the median were calculated and values less than the DL (<4 Bq m^−3^) were included in each quarter value. In addition, if a negative value was obtained due to statistical variation as a result of background subtraction, this value was assigned as a zero. The histogram for the mean annual indoor ^220^Rn concentrations is presented in Figure 1. The mean annual ^220^Rn concentration was found to vary from <4 to 383 Bq m^−3^. The arithmetic mean, the median, the geometric mean and the geometric standard deviation were 20.1 ± 36.8, 9.6, 10.0 Bq m^−3^ and 3.2, respectively. The ^222^Rn concentrations varied from 3.1 to 208 Bq m^−3^. The arithmetic mean, the median, the geometric mean and the geometric standard deviation were 15.5 ± 13.5, 11.7, 12.7 Bq m^−3^ and 1.78, respectively [23]. As a comparison, Kim et al. reported that the geometric mean for ^220^Rn concentrations in Korea was 10.7 Bq m^−3^. The log-normal cumulative frequency distribution for the indoor ^220^Rn concentrations is shown in Figure 2. The ^220^Rn concentration distribution would appear to be close to a log-normal distribution. The distribution of the mean annual indoor ^220^Rn concentrations was accepted as a log-normal distribution based on the Kolmogorov–Smirnov test at a significance level of 95%.

### 3.2. Seasonal Variation

The indoor ^220^Rn concentration data for each season are presented in Table 1. Negative values in this dataset were eliminated for calculation of the geometric mean. A significant seasonal variation in the ^220^Rn concentrations for the four seasons was not found. According to Kim et al. [15] and Stjanovska et al. [16], a temporal pattern in the ^220^Rn concentration data was observed with values in the winter and spring seasons being higher than those in the summer and autumn. Martinez et al. [17] found that the highest concentrations for Mexico City were in the autumn season and the lowest concentrations were in summer.

In the present study, slight differences were noted in the ^220^Rn concentrations depending on the periods of exposure. The lowest ^220^Rn concentrations for all types of houses were observed in the winter season (October–December). However, a different relationship was noted for the ^222^Rn concentrations, namely, that the ^222^Rn concentrations tended to be higher in winter compared to the other seasons [23]. This was probably because the residents used domestic heaters to maintain a comfortable room temperature in winter, and consequently there would have been increased ventilation rates due to the contribution of convection and/or stack effect in the rooms.

The variation of the ^220^Rn concentration in the rooms was slightly different from that of ^222^Rn, which may reflect the differences in the half-lives and sources of ^220^Rn, despite the fact that there were large fluctuations in the standard deviations for the seasonal variations of ^220^Rn concentrations. The reason why the indoor ^220^Rn concentrations did not display a variation similar to ^222^Rn is unclear at this time.

### 3.3. Nature of Housing

Indoor ^220^Rn concentrations were categorized in terms of the structural features of the housing. The annual mean, the standard deviation, and the geometric mean for the indoor ^220^Rn concentrations together with number of houses monitored are given in Table 2. The arithmetic and geometric mean concentrations for wooden and concrete-based houses have higher values than those of other structures, although there were large fluctuations in the data. The maximum value was found for a wooden house with a mud wall, the highest ^220^Rn concentration being 383 Bq m^−3^. The cause of the high ^220^Rn concentration of wooden houses is that they have relatively high ratio of the mud wall in comparison to other house structure types. Table 3 lists the ratio of the mud wall in each housing type. Accordingly, the ^220^Rn concentrations in wooden houses are higher than those for other housing types. 

With respect to the ^220^Rn concentrations by region, the overall ratios for wooden houses with mud walls in the Hokkaido—Tohoku, Kanto and Kyushu—Okinawa areas are lower than for those in other areas of Japan. Therefore, the ^220^Rn concentrations in these former areas also tends to be lower than the values found in the other areas.

### 3.4. Dependency of ^220^Rn Concentration on Wall Structure and Distance from Wall

**Figure 3:**
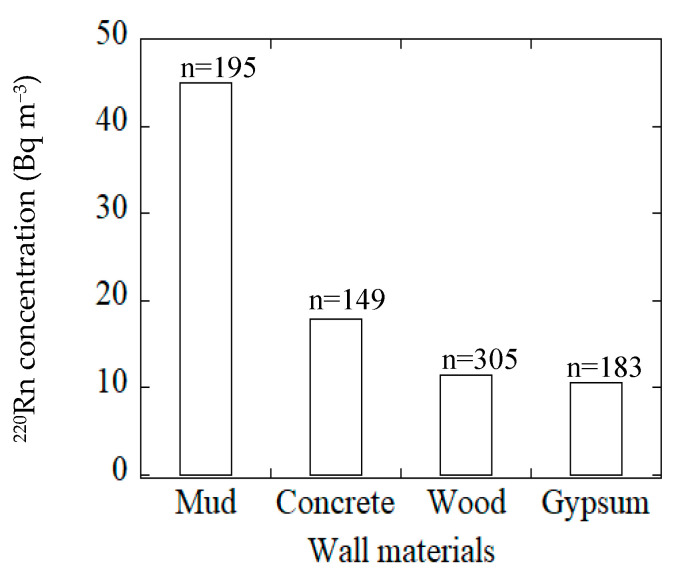
^220^Rn concentrations for various wall materials.

**Figure 4:**
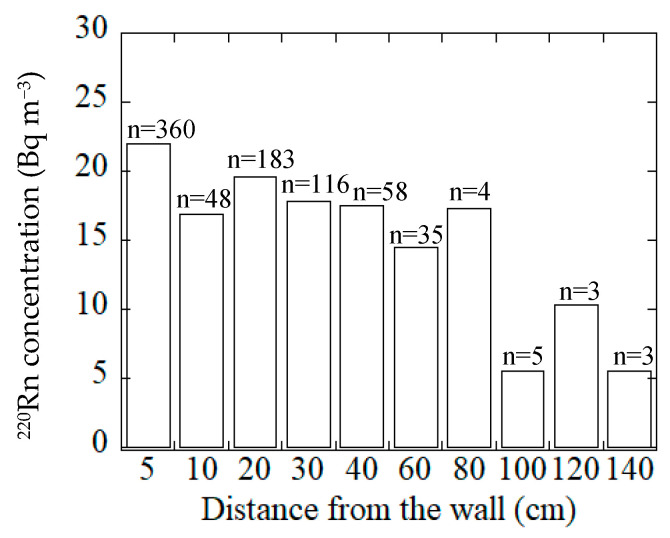
^220^Rn concentrations at different distances from the wall surface for all wall materials.

The present survey on ^220^Rn concentrations considered four categories of material which were used for wall construction in the houses. The mean annual ^220^Rn concentrations obtained by passive measurement for the different wall materials in the houses are presented in Figure 3. Inspection of the results (Figure 3) reveals that high ^220^Rn concentrations occurred for houses with mud walls, and the values decreased gradually with distance from the surface of the wall as shown in Figure 4. Yonehara et al. reported similar behavior for ^220^Rn concentrations at locations near the wall surfaces in Japanese dwellings [26].

### 3.5. ^220^Rn and ^222^Rn Correlation

**Figure 5:**
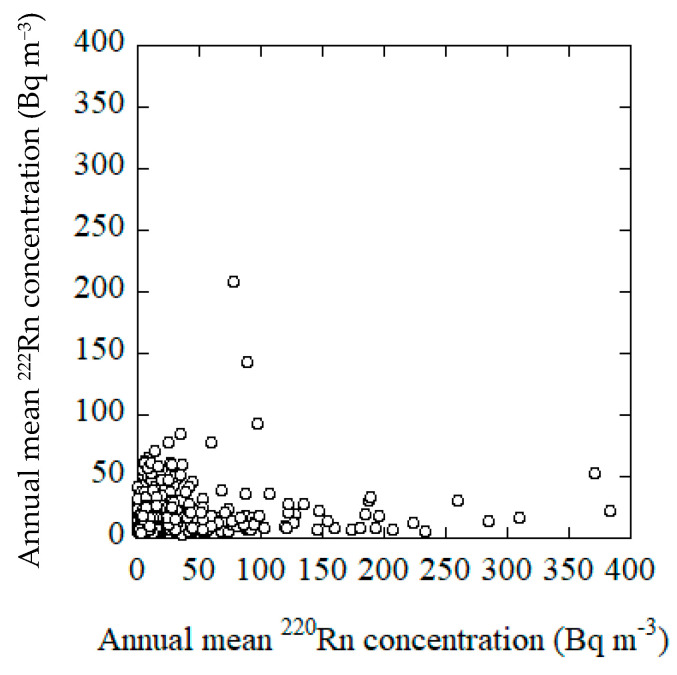
Correlation between the mean annual concentrations of ^220^Rn and ^222^Rn.

The correlation between the indoor ^220^Rn and ^222^Rn concentrations was investigated. The relationship between the ^220^Rn and the ^222^Rn concentrations is illustrated in Figure 5. The concentration distributions for both radioisotopes follow approximately a log-normal distribution. Consequently, both datasets were calculated after taking the logarithms of the respective data. The linear regression analysis shows a weak positive correlation (R = 0.25). The ratio for the concentrations of ^220^Rn/^222^Rn ranged from 0.007 to 40.3 and reveal a log-normal plot. The arithmetic mean for ^220^Rn/^222^Rn was 1.64 and geometric mean was 0.78.

## 4. Conclusions

The mean annual indoor ^220^Rn concentrations were measured in 899 houses using a passive ^222^Rn–^220^Rn discriminative monitor. The arithmetic mean, the median and the geometric mean were 20.1, 9.6 and 10.0 Bq m^−3^, respectively. The ^220^Rn concentration plot exhibited a log-normal distribution. The maximum ^220^Rn concentration found in the present survey was 383 Bq m^−3^ for a wooden house with mud walls. The survey data for the indoor ^220^Rn concentrations in Japan did not exhibit a significant seasonal variation. There was a marked difference in the ^220^Rn concentration depending on the nature of the house structure. Relatively higher concentrations of ^220^Rn were found in wooden and concrete block houses compared to other housing types. In general, the ^220^Rn concentrations in traditional wooden houses with mud walls tended to be higher than those for houses with different wall types. Further, it was demonstrated that the ^220^Rn concentrations decreased with distance of measurement from the wall.

## Figures and Tables

**Table 1:** ^220^Rn concentrations measured in the different seasons.

Measurement Period	Number of Houses	^220^Rn (Bq m^−3^)			GSD
		AM	SD	GM (Number of Houses)	
January–March	899	18.9	40.0	14.6 (576)	3.5
April–June		22.8	39.2	14.4 (733)	3.5
July–September		21.9	42.3	14.0 (713)	3.3
October–December		16.6	41.0	13.0 (492)	3.9

AM: Arithmetic mean; SD: Standard deviation; GM: Geometric mean; GSD: Geometric standard deviation (dimensionless).

**Table 2:** The mean annual ^220^Rn concentration for each type of house.

Structure	Number of Houses	^220^Rn (Bq m^−3^)		
		AM	SD	GM
Wooden	597	23.1	40.7	10.8
Concrete	182	16.3	32.5	9.6
Steel frame	90	8.6	8.9	6.1
Concrete block	16	21.8	25.6	13.8
Prefabricated	6	3.4	2.6	2.7

AM: Arithmetic mean; SD: Standard deviation; GM: Geometric mean.

**Table 3:** Ratio of mud wall in each structure type.

Structure Type	Total Number of Houses	Number of Mud Wall Houses	Ratio of Mud Wall in the House (%)
Wooden	597	190	31.8
Concrete	182	3	1.6
Steel frame	90	0	0
Concrete block	16	1	6.3
Prefabricated	6	0	0

## Data Availability

The data presented in this study are available on request from the corresponding author.
